# Significant Role of Estrogen and Progesterone Receptor Sequence Variants in Gallbladder Cancer Predisposition: A Multi-Analytical Strategy

**DOI:** 10.1371/journal.pone.0040162

**Published:** 2012-07-10

**Authors:** Anshika Srivastava, Kiran Lata Sharma, Neena Srivastava, Sanjeev Misra, Balraj Mittal

**Affiliations:** 1 Department of Genetics, Sanjay Gandhi Post Graduate Institute of Medical Sciences (SGPGIMS), Lucknow, India; 2 Department of Physiology, Chhatrapati Shahuji Maharaj Medical University (CSMMU), Lucknow, India; 3 Surgical Oncology, Chhatrapati Shahuji Maharaj Medical University (CSMMU), Lucknow, India; Ohio State University Medical Center, United States of America

## Abstract

**Background:**

Carcinoma of gallbladder (GBC) is an aggressive malignancy. The higher incidence of gallbladder cancer in women has been partly attributed to hormonal factors. Therefore the present study was designed to explore the role of genetic variants in estrogen (*ESR1, ESR2*) and progesterone (*PGR*) receptors in conferring risk of gallbladder cancer.

**Materials and Methods:**

The present case-control study recruited total of 860 subjects, including 410 GBC patients, 230 gallstone patients and 220 controls. We examined the associations of 6 selected polymorphisms in three genes: *ESR1* (rs2234693, rs9340799, rs1801132), *ESR2* (rs1271572, rs1256049) and *PGR* (rs1042838) with GBC risk. Genotyping for all the polymorphisms was done using PCR-RFLP. Multifactor dimensionality reduction and classification and regression tree approaches were combined with logistic regression to discover high-order gene-gene interactions in hormonal pathway.

**Results:**

On comparing the genotype frequency distribution in gallstone and GBC patients with that of healthy subjects, the homozygous variant genotypes of *ESR1*-397TT (rs2234693) polymorphism showed significant risk for developing gallstone [odds ratio: OR = 2.9] and GBC [OR = 1.8] respectively. Detailed haplotypes analysis suggested that *ESR1* T_ rs2234693_G_ rs9340799_C_ rs1801132_ have significant association in conferring risk for both gallstones [OR = 2.2] and GBC [OR = 3.0]. However, the variant-containing genotypes (DI+II) of *PGR* (rs1042838) showed low risk in both GBC [OR = 0.4] and gallstone patients [OR = 0.4].On performing the MDR analysis, *ESR1* IVS1-397C>T, *ESR1* IVS1-351A>G, and *ESR2*-789 A>C yielded the highest testing accuracy of 0.634. These results were further supported by the CART analysis which revealed that individuals with the combined genotypes of *ESR1*-397 CT or TT, *ESR1*-351 AG or GG and *ESR2* -789 AA had the highest risk for GBC [OR  = 3.9].

**Conclusion:**

Using multi-analytical approaches, our study showed important role of *ESR1* IVS1-397C>T, *ESR1* IVS1-351A>G, and *ESR2*-789 A>C variants in GBC susceptibility and the risk appears to be mediated through gallstone dependent pathway.

## Introduction

Carcinoma of the gallbladder is a highly fatal disease with late diagnosis, limited treatment options and deprived prognosis [1]. It is the most common malignant lesion of the biliary tract and the sixth most common among malignant neoplasms of the digestive tract [1], [2], [3]. A study from Utah cancer registry (UCR) and Swedish family-cancer database reported familial clustering of GBC [4]. Compelling evidence also exists for the role of family history of gallstones in gallbladder cancer etiology [5], [6]. Moreover, the incidence graph of gallbladder carcinoma fluctuates with sex and ethnicity and the highest frequencies are reported in females belonging to Native Americans, South America, and North India [7].

A multifaceted interplay between hormones, metabolic alterations, infections, and even anatomical anomalies have been elucidated in the etiology of gallbladder carcinoma [1]. Moreover, a plethora of epidemiological studies have shown strong association of GBC with cholesterol gallstone disease [8] and with many of its risk factors like obesity, high carbohydrate intake, and female sex [9]. In post menopausal women, hormone replacement therapy significantly increases the risk of gallbladder diseases [10], [11] suggesting a noteworthy role of sex hormones in the etiology of GBC [12], [13], [14], [15], [16].Also women are two to six times more affected than men. Altogether, these evidences have raised the possibility that sex steroids (estrogen and progesterone) could play a key pathophysiological role in the development of gallbladder carcinoma. The estrogen and progesterone hormones act on target tissues by binding to their respective receptors. Estrogen receptors (*ESR1, ESR2*) and progesterone receptor (*PGR)* genes are located on chromosome 6q25.1, 14q21–22 and 11q22 respectively and their expression have been detected in human gallbladder normal mucosa [15] and GBC [16] Allelic variants of estrogen receptor genes have been shown to be associated with susceptibility or progression with various disorders such as myocardial infarction [17], cholesterol gallstone and biliary tract diseases [18]. Moreover, functional assays suggest that *ESR1* IVS1-397C>T polymorphism affects a binding site for the myb family of transcription factors [19], [20] and the polymorphism has also been studied in various breast cancer association studies [17], [21], [22]. On the contrary, hormone progesterone plays an important role in regulating the level of estrogen and providing protection against several cancers [23], [24].

Given the potential hormonal role in gallbladder diseases, and also the previously explored role of *ESR/PGR* polymorphisms in female related cancers, we hypothesized that genetic variants in *ESR1*, *ESR2* and *PGR* genes may have significant impact on the risks of gallbladder cancer. Therefore, in the present study, we investigated a panel of 6 well-studied polymorphisms in *ESR1, ESR2* and *PGR* genes in a case–control design involving 410 GBC patients, 230 gallstone patients and 220 cancer/gallstone-free controls from North India. In addition to logistic regression (LR), two non-parametric approaches, multifactor dimensionality reduction (MDR) and classification and regression tree (CART) were applied to explore high-order gene–gene interactions in modulating risk of gallbladder cancer. To the best of our knowledge, this is the first report that has investigated the role of genetic variants in hormonal receptor genes using multi-analytic approach to define individual risk profiles for gallbladder cancer and gallstone disease.

## Results

### Population Characteristics

The demographic characteristics of GBC and gallstone patients with respect to their age and gender matched controls are presented in Table 1. The mean age in all the three groups were comparable and demonstrated no statistically significant differences. More than 90% of the GBC patients were in advanced stages of cancer (stages III and IV) and gallstones were found to be present in 50.5% of GBC patients. About 31% of the GBC patients were associated with tobacco usage in some form (smoking, chewing, or both). It was observed during data collection that majority of the female patients were housewives and male patients were not engaged in any hazardous occupations. All cancer and gallstone patients were incident cases, and none of the controls had family history of cancer.

**Table 1:** Demographic Profile of the Study Subjects.

Variables	GBC Patients (GBC)	Gallstone Patients (GS)	Controls
	N (%)	N (%)	N (%)
Subjects	410	230	220
Sex – no%			
Female	288(70.2)	156 (67.8)	132 (60.0)
Male	122(29.8)	74 (32.2)	88 (40.0)
Age ± SD	52.32±10.6	48.65±12.39	52.0±9.6
Stages			
0, I	None		
II	24(5.8)		
III	201 (49.0)		
IV	185 (45.2)		
Gallstone present – no%	207 (50.5)	230 (100)	None
Gallstone absent – no%	203 (49.5)	None	220 (100)
Tobacco users –	123 (31%)	–	–
Tobacco nonusers –	274 (69%)	–	–
Early age of onset-	150 (36.6)	–	–
Late age of onset-	260 (63.4)	–	–

### Allelic Distribution of Studied Polymorphisms in Controls

The genotypic and allelic distribution of *ESR1* IVS1-397C>T (rs2234693), IVS1-351A>G (rs9340799), Ex4-122C>G (rs1801132), *ESR2* -789 A>C (rs1271572), 1082 G>A (rs1256049) and *PGR* ins/del (rs1042838) are shown in Table 2. The observed genotype frequencies of all the studied polymorphisms in controls were in accordance with Hardy-Weinberg equilibrium (p<0.05). The frequencies of the variant alleles in our study were similar to previous published reports [25].

**Table 2:** Overall Frequency Distribution of *ESR1, ESR2* and *PGR* Polymorphisms in GBC, GS Patients and Healthy Subjects.

Genotype/Allele	Controls n(%)	GBCn(%)		*p*-value^a^	OR(95%CI)^a#^	GSn(%)	*p*-value^b^	OR(95%CI)^ b#^
***ESR 1*** ** IVS1-397C>T**								
***CC***	91 (41.4)		133 (32.4)	–	1(Reference)	64 (27.8)	–	1(Reference)
***CT***	110 (50.0)		218 (53.2)	0.06	1.4 (0.9–2.1)	128 (55.7)	**0.019**	1.6 (1.0–2.5)
***TT***	19 (8.6)		59 (14.4)	**0.02**	1.8 (0.9–3.4)	38 (16.5)	**0.001**	2.9 (1.5–5.7)
***CT+TT***	129 (58.6)		277 (67.6)	**0.03**	1.5 (1.0–2.1)	166 (72.2)	**0.003**	1.8 (1.2–2.5)
***C***	292 (66.4)		484 (59.0)	–	1(Reference)	256 (55.6)		1(Reference)
***T***	148 (33.6)		336 (41.0)	**0.02**	1.2 (0.9–1.6)	204 (44.3)	**0.001**	1.5 (1.1–2.1)
***ESR1*** ** IVS1-351A>G**								
***AA***	90 (40.9)		140 (34.1)	–	1(Reference)	69 (30.0)	–	1(Reference)
***AG***	109 (49.5)		205 (50.0)	0.47	1.1 (0.7–1.6)	117 (50.9)	0.14	1.3 (0.9–2.0)
***GG***	21 (9.5)		65 (15.9)	0.09	1.6 (0.9–3.0)	44 (19.1)	**0.002**	2.6(1.4–4.9)
***AG+ GG***	130 (59.1)		271 (66.1)	0.24	1.2 (0.8–1.8)	161 (70.0)	**0.02**	1.5 (1.0–2.3)
***A***	289 (65.6)		485 (59.1)	–	1(Reference)	255 (55.4)	–-	1(Reference)
***G***	151 (34.3)		335 (40.9)	0.14	1.2 (0.9–1.6)	205(44.6)	**0.005**	1.4 (1.1–1.9)
***ESR1*** ** Ex4-122C>** ***G***								
***CC***	106 (48.2)		199 (48.5)	–	1(Reference)	120 (52.2)	–	1(Reference)
***CG***	104 (47.3)		184 (44.9)	0.78	0.9 (0.6–1.3)	97 (42.2)	0.48	0.8 (0.5–1.2)
***GG***	10 (4.5)		27 (6.6)	0.46	1.3 (0.6–3.0)	13 (5.7)	0.98	0.9 (0.4–2.4)
***CG+GG***	114 (51.8)		211 (51.5)	0.93	0.9 (0.6–1.4)	110 (47.8)	0.51	0.8 (0.6–1.2)
***C***	316 (71.8)		582(71.0)	–	1(Reference)	338 (73.5)	–	1(Reference)
***G***	124 (28.2)		238 (29.0)	0.92	1.0 (0.7–1.3)	122 (26.5)	0.30	0.9 (0.7–1.2)
***ESR2*** ** -789 A>C**								
***AA***	94 (42.7)		177 (43.2)	–	1(Reference)	105 (45.7)	–	1(Reference)
***AC***	109 (49.5)		207 (50.5)	0.68	1.0 (0.7–1.5)	107 (47.0)	0.59	0.7 (0.3–1.5)
***CC***	17 (7.7)		26 (6.3)	0.46	0.7 (0.3–1.5)	18 (7.3)	0.72	0.7 (0.3–1.8)
***AC+CC***	126 (57.3)		233 (56.8)	0.63	0.9 (0.6–1.2)	125 (55.4)	0.57	0.8 (0.5–1.3)
***A***	297 (67.5)		561 (68.4)	–	1(Reference)	317 (68.9)	–	1(Reference)
***C***	143 (32.5)		259 (31.6)	0.59	0.6 (0.4–3.1)	143 (31.0)	0.52	0.6 (0.2–1.9)
***ESR2*** ** 1082 G>A**								
***GG***	206 (93.6)		385 (93.9)	–	1(Reference)	212 (92.2)	–	1(Reference)
***GA+ AA***	14 (6.4)		25 (6.1)	0.86	0.9 (0.4–2.0)	18 (7.8)	0.59	1.2 (0.5–2.5)
***G***	426 (96.8)		795 (97.0)	–	1(Reference)	442 (96.1)	–	1(Reference)
***A***	14 (3.2)		25 (3.0)	0.79	1.0 (0.6–2.0)	18 (3.9)	0.41	1.3 (0.6–2.8)
***PGR*** ** Ins/Del**								
***DD***	181 (82.3)		368 (89.8)	–	1(Reference)	208 (90.4)	–	1(Reference)
***DI***	37 (16.8)		42 (10.2)	**0.009**	0.5 (0.3–0.8)	22 (9.6)	**0.001**	0.5 (0.2–0.9)
***II***	2 (0.9)		0	–	–	0	–	–
***DI+II***	39 (17.7)		42 (10.2)	**0.004**	0.4 (0.2–0.7)	22 (9.6)	**0.009**	0.4 (0.2–0.7)
***D***	399 (90.7)		778 (94.9)	–	1(Reference)	438 (95.2)	–	1(Reference)
***I***	41 (9.3)		42 (5.1)	**0.002**	0.4 (0.3–0.8)	22 (4.8)	**0.002**	0.5 (0.3–0.9)

GBC-Gallbladder cancer, OR -Odds Ratio, CI-Confidence Interval;

a =  represents the p value and odds ratio for the comparison of wild, heterozygous and variant genotypes and alleles in.

Healthy Controls (HC) v/s GBC;

b =  represents the p value for the comparison of wild, heterozygous and variant genotypes and alleles in HC v/s Gallstones.

Significant values are in bold.

# Data calculated by LR, adjusted for sex and age status.

### Association of *ESR1*, *ESR2* and *PGR* Polymorphisms with GBC and Gallstones

Table 3 shows the risk of gallbladder carcinoma and stones in relation to each of the SNPs of *ESR1, ESR2* and *PGR.* On comparing the genotype frequency distribution of our study groups i.e. GBC and gallstone patients with that of controls, the homozygous variant genotypes of *ESR1* IVS1-397C>T (rs2234693) polymorphism showed statistically significant increased risk for developing GBC (p = 0.02; [OR], 1.8) and gallstone (p = <0.001; [OR], 2.9). Furthermore, in gallstone patients *ESR1*IVS1-351A>G conferred higher risk (p = 0.002; [OR], 2.6). On the contrary, no significant differences were observed in the distribution of *ESR1* IVS1-351A>G, Ex4-122C>G, (rs1801132) and *ESR2* -789 A>C (rs1271572), Ex6 1082 G>A (rs1256049) polymorphisms in any of the groups, both at genotypic and allelic levels. The variant-containing genotypes (DI+II) of *PGR* ins/del (rs1042838) showed low risk in both GBC and gallstone patients which was also significant (p = 0.004; [OR], 0.4; p = 0.009; [OR], 0.4 Table 2) when compared with homozygous wild-type DD genotype.

**Table 3:** Frequency Distribution *ESR1* and *PGR* Polymorphisms after Subdividing on the Basis of Gallstone Status.

Genotypes/Alleles	Controlsn (%)	GBC without gallstone	GBC with gallstone	*p-*value^a^	OR^a^ (95%CI)	*p*-value^b^	OR^b^ (95% CI)
***ESR 1*** ** IVS1-397C>T**							
***CC***	91 (41.4)	71 (35.0)	62 (30.0)	–	1(Reference)	–	1(Reference)
***CT***	110 (50.0)	102 (50.2)	116 (56.0)	0.39	1.2 (0.7–1.8)	**0.03**	**1.6 (1.0–2.6)**
***TT***	19 (8.6)	30 (14.8)	29 (14.0)	0.10	1.7 (0.8–3.4)	0.14	1.7 (0.8–3.6)
***CT+TT***	258 (58.6)	264 (65.0)	290 (70)	0.08	1.2 (0.9–1.7)	**0.002**	**1.6 (1.2–2.3)**
***C***	292 (66.4)	244 (60.1)	240 (58)	–	1(Reference)	–	1(Reference)
***T***	148 (33.6)	162 (39.9)	174 (42)	0.28	1.1 (0.8–1.5)	0.07	1.3 (0.9–1.8)
***ESR1*** ** IVS1-351A>G**							
***AA***	90 (40.9)	69 (34.0)	71 (34.3)		1(Reference)	–	1(Reference)
***AG***	109 (49.5)	103 (50.7)	102 (49.3)	0.47	1.1 (0.7–1.8)	0.81	1.0 (0.6–1.7)
***GG***	21 (9.5)	31 (15.3)	34 (16.4)	0.15	1.6 (0.8–3.2)	0.16	1.6 (0.8–3.3)
***AG+GG***	260 (59.1)	270 (66.5)	272 (65.7)	0.14	1.2 (0.9–1.6)	0.37	1.1 (0.8–1.6)
***A***	289 (65.7)	244 (60.1)	244 (58.9)	–	1(Reference)	–	1(Reference)
***G***	151 (34.3)	162 (39.9)	170 (41.1)	0.34	1.1 (0.8–1.5)	0.16	1.2 (0.9–1.7)
***ESR1*** ** Ex4-122C>G**							
***CC***	106 (48.2)	99 (48.8)	100 (48.3)	–	1(Reference)	–	1(Reference)
***CG***	104 (47.3)	92 (45.3)	92 (44.4)	0.81	1.0 (0.6–1.6)	0.36	0.8 (0.5–1.3)
***GG***	10 (4.5)	12 (5.9)	15 (7.2)	0.49	1.3 (0.5–3.5)	0.88	1.1 (0.4–2.7)
***CG+GG***	228 (51.8)	208 (51.2)	214 (51.7)	0.35	0.8 (0.6–1.1)	0.26	0.8 (0.6–1.1)
***C***	316 (71.8)	302 (74.4)	292 (70.5)	–	1(Reference)	–	1(Reference)
***G***	124 (28.2)	104 (25.6)	122 (29.5)	0.83	0.9 (0.6–1.4)	0.56	0.9 (0.6–1.2)
***PGR*** ** Ins/del**							
*DD*	181 (82.3)	186 (91.6)	182 (87.9)		1(Reference)		1(Reference)
*DI+II*	37 (16.8)	17 (8.4)	25 (12.1)	**0.005**	0.3 (0.2–0.7)	0.07	0.5 (0.3–1.0)
*II*	2 (0.9)	0	0	–	–	–	–
*DI+II*	78 (17.7)	34 (8.4)	50 (12.1)	**0.001**	0.3 (0.2–0.5)	**0.005**	0.5 (0.3–0.8)
*D*	399 (90.7)	389 (95.8)	389 (94)		1(Reference)		1(Reference)
*I*	41 (9.3)	17 (4.2)	25 (6)	**0.002**	0.3 (0.2–0.7)	0.11	0.6 (0.3–1.1)

a =  represents the p value and odds ratio for the comparison of wild, heterozygous and variant genotypes and alleles in Healthy Controls (HC) v/s GBC without gallstone patients b =  represents the p value for the comparison of wild, heterozygous and variant genotypes and alleles in Healthy Controls (HC) v/s GBC with gallstone patients.

Significant values are in bold.

The study subjects were stratified in males and females. In male GBC patients, the variant genotype TT of *ESR1* IVS1-397C>T (rs2234693) polymorphism showed significantly increased risk for GBC (p = 0.009; [OR], 4.8) whereas IVS1-351A>G (rs9340799) polymorphism conferred increased risk for GBC in females (p = 0.026; [OR], 2.4). These conflicting results may partly be due to inadequate number of males compared to female GBC patients. None of the genetic variants of *ESR2* and *PGR* ins/del conferred gender-specific risk for GBC both at genotypic and allelic levels. Similar results were obtained when we compared gallstone males and females with respective sex segregated controls. (Data not shown.).

### *ESR1* and *PGR* Polymorphisms and Modulation of Risk in the Presence of Gallstones

Since gallstones are present in more than 50% of GBC patients, the cancer cases were segregated into two groups on the basis of presence or absence of accompanying gallstones and compared independently with controls (Table 3). GBC patients with accompanying gallstones were found to have higher risk of developing the disease with *ESR1* IVS1-397 CT+TT (rs2234693) genotypes (p = 0.002; [OR], 1.6 Table 3). In contrast, *ESR1* Ex4-122C>G (rs1801132) was not found to be significantly associated in both the subgroups. In case of *PGR* ins/del (rs1042838), a protective effect was observed in GBC patients irrespective of their gallstone status (Table 3). However, on comparing the GBC patients having gallstones with gallstone patients (no cancer), the results showed no association with all studied polymorphisms of *ESR1, ESR2* and *PGR,* both at genotypic and allelic levels. (Data not shown.).

### Linkage Disequilibrium and Haplotypes Analysis of *ESR1* in Case and Control Groups

On LD analysis, *ESR1* rs2234693 and rs9340799 were found to be in linkage disequilibrium (D’ = 0.575). Haplotypes were constructed for the three polymorphisms in *ESR1* gene including IVS1-397C>T (rs2234693); IVS1-351A>G (rs9340799) and Ex4-122C>G (rs1801132). The haplotypes comprising the homozygous wild alleles were taken as reference and the difference in the frequencies of haplotypes between patients and controls were tested using chi-square test.

Haplotypes analysis of the studied three polymorphisms of *ESR1* revealed that distribution of T_ rs2234693_G_ rs9340799_C_ rs1801132_ haplotype was significantly higher in both GBC (27.5% v/s 13.7%) and gallstone patients (25.1% v/s 13.7) in comparison to controls and was conferring high risk for GBC (p = <0.0001; [OR], 3.0 Table 4) and gallstone disease (p = 0.0012; [OR], 2.2 Table 4). Global haplotypes analysis indicated a statistically significant difference between GBC cases and controls based on the distribution pattern of the *ESR1* haplotypes (p = <0.001). However, none of the *ESR2* haplotypes were found to be associated with GBC and gallstone risk.

**Table 4:** Frequency distribution of haplotypes of *ESR1* Polymorphisms in Study Subjects.

Gallbladder cancer patients and Controls						
Haplotypes	Frequency				*p-* value	OR (95%CI)
	GBC (%)		HC (%)			
C _rs2234693_ A _rs9340799_C _rs1801132_	–		0.2879		0.3783	
T_ rs2234693_G_ rs9340799_C_ rs1801132_	0.275		0.1371		**<0.0001**	**3.04 (1.83–5.04)**
C_ rs2234693_A_ rs9340799_G_ rs1801132_	0.1513		0.1437		0.3	1.36 (0.76–2.43)
C_ rs2234693_G_ rs9340799_C_ rs1801132_	0.0917		0.101		0.33	1.35 (0.74–2.48)
T_ rs2234693_A_ rs9340799_C_ rs1801132_	0.0841		0.1018		0.48	1.24 (0.69–2.24)
T_ rs2234693_G_ rs9340799_G_ rs1801132_	0.0818		0.0643		**0.047**	**2.06 (1.01–4.21)**
C_ rs2234693_G_ rs9340799_G_ rs1801132_	0.0165		0.0407		0.38	0.54 (0.14–2.14)
T_ rs2234693_A_ rs9340799_G_ rs1801132_	0.0117		0.0331		0.4	0.54 (0.13–2.30)
**Global haplotype association p-value: <0.0001**						
**Gallstone patients and Controls**						
		**GS (%)**		**HC (%)**		
C _rs2234693_ A _rs9340799_C _rs1801132_		0.334		0.3783	–	1.00
T _rs2234693_G _rs9340799_C _rs1801132_		0.251		0.1371	**0.0012**	**2.22 (1.37–3.59)**
C _rs2234693_A _rs9340799_G _rs1801132_		0.1034		0.1437	0.61	0.84 (0.44–1.62)
C _rs2234693_G r_s9340799_C _rs1801132_		0.0831		0.101	0.7	1.13 (0.62–2.04)
T _rs2234693_A _rs9340799_C _rs1801132_		0.0645		0.1018	0.52	0.78 (0.37–1.66)
T _rs2234693_G _rs9340799_G _rs1801132_		0.0755		0.0643	0.2	1.56 (0.79–3.07)
C _rs2234693_G _rs9340799_G_ rs1801132_		0.0524		0.0331	0.18	1.91 (0.75–4.86)
T _rs2234693_A _rs9340799_G _rs1801132_		0.036		0.0407	0.89	0.94 (0.38–2.34)
**Global haplotype association p-value: 0.0042**						

Significant values are in bold.

### Gene–gene Interaction

As there may be significant interactions between *PGR, ESR1* and *ESR2*, overall gene- gene interaction analysis was performed. The results shown in Table 5 revealed significant interaction in specific variants of the three genes with overall interaction p value <0.0001.

**Table 5:** Overall interaction of studied variations (*PROGIN, ESR1, ESR2*).

Combined Alleles	*p-* value	OR (95%CI)
D_rs1042838_C_rs2234693_A_rs9340799_C_rs1801132_A_rs1271572_G_rs1256049_	–	1.00
D_rs1042838_T_rs2234693_G_rs9340799_C_rs1801132_A_rs1271572_G_rs1256049_	**0.04**	**2.3(1.03–5.33)**
D_rs1042838_T_rs2234693_G_rs9340799_G_rs1801132_A_rs1271572_G_rs1256049_	**0.04**	**9.4 (1.08–83.42)**
D_rs1042838_C_rs2234693_G_rs9340799_C_rs1801132_C_rs1271572_G_rs1256049_	**0.03**	**0.2 (0.06–0.92)**

Significant values are in bold.

### Association of High-order Interactions with GBC Risk by MDR Analysis

Our earlier results have shown the involvement of high order gene-gene interactions in DNA repair and inflammatory pathways in GBC susceptibility [26]. Therefore, we looked for such interactions in the genetic variants of hormonal receptor genes, using MDR and CART analysis.

Table 6 shows the best interaction model by MDR analysis. The best one-factor model for predicting GBC risk was *ESR1* IVS1-397C>T SNP (testing accuracy  = 0.519, CVC  = 10/10, permutation p  = 0.025). The best two-factor model of *ESR1* IVS1 351A>G and *ESR2* -789 A>C had an improved testing accuracy of 0.564 (permutation p = <0.001), however, the CVC were decreased (6/10). The best interaction model was the three-factor model including *ESR1* IVS1-397C>T *ESR1* IVS1 351A>G and *ESR2* -789 A>C SNPs, which yielded the highest testing accuracy of 0.634 and the maximal CVC of 10/10 (permutation p = <0.001). The four-factor model consisting of *ESR1* IVS1-397C>T, IVS1 351A>G, Ex4-122C>G and *ESR2* -789 A>C also improved testing accuracy compared with the one-factor model (CVC  = 10/10 permutation p  =  <0.001). For the three SNPs identified in the best interaction model, *ESR1* IVS1-397C>T, IVS1 351A>G and *ESR2*-789 A>C were combined and dichotomized according to the MDR software. Individuals carrying the combined risk stratum had a 4.0 fold increased risk for GBC (p = <0.001). Furthermore, a combined effect of *ESR1* IVS1-397C>T, IVS1 -351A>G and *ESR2* -789 A>C was evaluated by logistic regression analysis (Table 7) with the *ESR1* IVS1-397TT, IVS1-351GG and *ESR2* -789 AA as risk genotypes. Subjects were categorized into four groups based on the number of risk genotypes they carried and those without any risk genotype were designated as the reference group. We found that the p-values for individuals carrying one and two risk genotypes was 0.46 and 0.015 respectively, However, the p value and ORs for the three risk genotypes could not be ascertained because of the absence of the variant combination in the controls (Table 7). These results suggest a significant gene dosage effect of *ESR1* IVS1-397C>T, IVS1 -351A>G and *ESR2* -789 A>C.

**Table 6:** Interaction models by MDR analysis.

No. of Risk Factors	Best Interaction Model	Testing Accuracy	#CVC	*P* for permutation Testing
1	*ESR1 IVS1-397C>T*	0.519	8/10	0.025
2	*ESR1 IVS1 351A>G, ESR2* -789 A>C	0.564	6/10	<0.001
3	a *ESR1 IVS1-397C>T ESR1 IVS1 351A>G, ESR2* -789 A>C	0.634	10/10	<0.001
4	*ESR1 IVS1-397C>T ESR1 IVS1 351A>G, ESR1 Ex4-122C>G* *ESR2* -789 A>C	0.591	10/10	<0.001

# CVC: Cross Validation Consistency.

a The model with the maximum testing accuracy and maximum CVC cross was considered as the best model.

The present study calculated, the best interaction model as the three-factor model including *ESR1 IVS1-397*C>T.

*ESR1 IVS1 351*A>G and *ESR2*-789 A>C polymorphisms.

**Table 7:** Combined effect of *ESR1* -397C>T, *ESR1* 351A>G and *ESR2* -789 A>C on GBC risk.

Number of risk genotypes	Cases	Controls	OR (95% CI)	p - value
	N (%)	N (%)		
0 (Cluster 1)	188 (45.9)	100 (45.5)	1 Reference	–
1 (Cluster 2)	158 (38.5)	108 (49.0)	0.8 (0.5–1.3)	0.15
2 (Cluster 3)	49 (12.0)	12 (5.5)	**2.1 (1.2–4.2)**	**0.025**
3 (Cluster 4)	15 (3.6)	0	–	–

The *ESR1 IVS1-397 TT, ESR1 IVS1 351GG* and *ESR2*-789 AA genotypes were considered as risk genotypes.

Cluster 1 individuals carried no risk genotypes; whereas in the next three groups, we pooled all individuals carrying.

risk genotype in any one gene (Cluster 2), in any two genes (Cluster 3) and all the three genes (Cluster 4).

### Association of High-order Interactions with GBC Risk by CART Analysis

The final resulting tree was generated by the CART analysis (Table 8). Consistent with the MDR best one-factor model, the initial split of the root node on the decision tree was *ESR1* IVS1-397C>T, suggesting that this SNP is the strongest risk factor for GBC among the polymorphisms examined. Further inspection of the tree structure revealed distinct interaction patterns between individuals carrying the *ESR1* IVS1-397 CT or TT and those with the *ESR2* -789 AC or CC genotypes. Individuals carrying *ESR1* IVS1-397CC, *ESR1*-122CC and *ESR2* -789 AC or AA genotypes had the lowest case rate of 33.3%, and taken as reference. Using the terminal node comprising the *ESR1* Ex4–122 CC genotype carriers as the reference, individuals carrying both the *ESR1* -397 CT or TT, *ESR1*-351 AG or GG and *ESR2* -789 AC genotypes exhibited a significantly higher risk for GBC (adjusted OR 3.6; 95% CI, 1.7–9.1), whereas individuals with the combined genotypes of *ESR1* -397 CT or TT, *ESR1* -351AG or GG, *ESR2* -789 AA had the highest risk for GBC (adjusted OR 3.9; 95% CI, 2.0–9.8) (Table 8). Thus, combining the single locus analysis, CART and MDR we found that single genetic variants in either *ESR1* or *ESR2* may not be responsible in conferring high risk for disease but rather a higher order gene-gene interactions are likely to be involved in genetic susceptibility to GBC.

**Table 8:** Risk estimates of CART terminal nodes.

Nodes	Genotypes	Cases	Controls	Case rate^a^ (%)	*p-*value	OR^b^ (95% CI)
1	*ESR1-397(CC) ESR2 -789 (AC/AA) ESR1 -122 (CC)*	17	34	33.3	–	1 Reference
2	*ESR1-397(CC) ESR2-789 (AC/AA) ESR1 -122 (CG)*	25	40	38.46	**0.048**	**1.3 (1.0–2.2)**
3	*ESR1-397(CT/TT) ESR1 -351 (AG/GG) ESR2 -789(AC)*	109	58	65.2	**1.3X10^−7^**	**3.6 (1.7–9.8)**
4	*ESR1-397 (CT/TT) ESR1 -351(AG/GG) ESR2 -789(AA)*	50	21	70.4	**1.5X10^−8^**	**3.9 (2.0–6.5)**
5	*ESR1 -397 (CT/TT) ESR1 351 (AG/GG) ESR2 -789 (AA)* *ESR1 -122 (GC/CC)*	77	17	81.9	**<0.001**	**3.0 (1.5–5.7)**

a Case rate is the percentage of cancer patients among all individuals in each node.

b ORs of terminal nodes were calculated by LR analysis adjusted for age and gender.

### In-silico Analysis of Genetic Variants on Gene Activity

As the SNPs are located in non-coding sequences, it was plausible that the SNPs may have influence on transcription of the gene. In-silico analysis using FAST-SNP and F-SNP showed change in transcriptional regulation and alternate splicing for most of selected SNPs (Table 8). For in-silco analysis of PROGINS, Polyphen” result showed the change as Benign whereas SIFT suggested “tolerated” change (Table 9).

**Table 9:** Results of F-SNP and FAST: SNP for all the studied polymorphisms.

Result of F SNP					Result of FAST- SNP	
Genetic Variation	Functional Category	Prediction Tool	Prediction Result	FS score	Possible Functional Effects	Risk
*ESR1 IVS1-397C>T* (rs2234693)	Transcriptional regulation	TFSearch	**Change**	**0.208**	Intronic with no known function	Unknown-Unknown (0–0)
		Consite	**Change**			
*ESR1 IVS1-351A>G* (rs9340799)	Transcriptional regulation	TFSearch	**Change**	**0.176**	Intronic enhancer	Very Low-Low(1–2)
*Ex4-122C>G* (rs1801132)	Protein coding	Ensembl-NS	**Synonymous**	**0.09**	Sense/ synonymous; Splicing regulation	Low-Medium (2–3)
	Splicing regulation	ESEfinder	**Change**			
*ESR2 -789 A>C* (rs1271572)	Transcriptional regulation	TFSearch	**Change**	**0.5**	Upstream with no known function	Unknown-Unknown (0–0)
*1082 G>A* (rs1256049)	Protein coding	Ensembl-NS	**Change**	**0.27**	Sense/ synonymous; Splicing regulation	Low-Medium (2–3)
		ESRSearch	**Change**			
		RESCUE_ESE	**Change**			
*PGR* (rs1042838)	Protein coding	PolyPhen	**Benign**	**0.5**	Intronic with no known function	Unknown-Unknown (0–0)
		SIFT	**Tolerated**			
		LS-SNP	**Benign**			
*PGR* (rs1042838)	Splicing regulation	Ensembl-NS	**Non** **Synonymous**	**0.5**	Intronic with no known function	Unknown-Unknown (0–0)
		ESEfinder	**Change**			
		ESRSearch	**Change**	**0.5**	Same as above	0–0

## Discussion

Genetic differences in sex hormone genes may have an effect on their respective activities, thereby causing inter-individual divergence in the propensity to GBC. Also, the strong female incidence has raised the likelihood that estrogens may play a key pathophysiological role in the progression of gallbladder cancer [27]. In addition, **e**xpression and functional studies have shown direct interactions between ESR and PGR receptor domains [28], [29]. There is evidence that inherent risk of GBC from cholelithiasis in patients without a family history of gallstones had a 21-fold risk, while those with both gallstones and a positive family history had a 57-fold higher risk [6].

In the present study, we applied a multi-analytic strategy combining LR, MDR and CART approaches to systematically examine the associations between GBC risk and a panel of genetic polymorphisms involved in hormonal pathway.

In the single-locus analysis, *ESR1* IVS1-397C>T (rs2234693) polymorphism showed significant association with GBC risk. Our results from LR, MDR and CART analyses also consistently suggested that *ESR1* IVS1-397C>T polymorphism is the most important single susceptibility factor for GBC development. Moreover, gene - gene interaction analysis showed significant interactions between these hormonal variants. In addition genethe multi-analytic strategies also revealed higher-order gene–gene interactions among *ESR1* IVS1-397C>T, IVS1 -351A>G and *ESR2* -789 A>C polymorphisms in GBC risk.

On performing detailed analysis of the haplotypes, we found that the gallbladder carcinoma and gallstones subjects who carry *ESR1* haplotypes IVS1-397T, IVS1-351G, Ex4-122C conferred increased risk for both GBC and gallstones indicating that *ESR1* haplotype as a risk factor. Thus, carriers of *ESR1* haplotypes had a 3.04 times increased risk of gallbladder carcinoma compared with non-carriers, whereas the same haplotypes conferred 2.2 times increased risk for gallstone diseases. In contrast, *Alu* insertion polymorphism of progesterone receptors (*PGR*) conferred lower risk in GBC and gallstone patients.

Recently, a study by Park et al. in Chinese population have evaluated the variations in hormone receptors (*ESR1, ESR2*) in relation to biliary tract cancers (35) and reported an association with *ESR1* (rs1801132) and *ESR2* (rs1255953) variants in GBC risk, but we did not observe any significant association with these exonic polymorphisms. Instead, we found association with the intronic (*ESR1*) and promoter (*ESR2*) polymorphisms. The reason behind this discrepancy could be the population variation. The allelic frequencies of *ESR1* and *ESR2* polymorphisms were not comparable between the two studies. Also, the total number of GBC cases enrolled by Park et al was relatively smaller as compared to the present study which raises the chances of Type II β error in their study. It is also possible that the SNPs of *ESR1* and *ESR2* may not be conferring direct effects on GBC susceptibility and their effects may be mediated through their linkage to some key functional polymorphisms.

The association between *ESR1* polymorphisms and risk of GBC/gallstones are biologically credible. The animal studies have shown that ESRs are present in the hepato-pancreatic-biliary tree [30], [31], [32] including bile duct epithelial cells and gallbladder, suggesting that estrogens may play a role in gallbladder diseases. In addition, immunohistochemical and quantitative RT PCR studies have also revealed that the expression level of *ESR1* gene is approximately 50 fold higher compared to *ESR2* [33] In animal models, 17beta estradiol promoted gallstone formation involves upregulation of hepatic expression of ERalpha but not ERbeta, and the lithogenic actions of estrogen can be blocked completely by the antiestrogenic agents ICI 182,780 [34]. These studies show that ESR-1 is key player and findings may offer a new approach to treat gallstones and gallbladder cancer by inhibiting hepatic ER activity with a liver-specific, ERalpha-selective antagonists. Some studies have highlighted the significant role of *ESR-2* rs1271572 in the risk of ovarian cancer [35], [36]. Moreover a study by MARIE-GENICA Consortium suggested that higher risk were observed in subjects having combined genotypes of both *ESR1* and *ESR2* genes which modified risk associated with estrogen monotherapy used in breast cancer [37]. Similarly, in our study, applying gene-gene interactions and multianalytical approaches revealed that GBC risk was higher when the variant genotypes of *ESR1* and wild *ESR2* -789 A>C were present in combinations.

It may also be mentioned that genetic variants in several other genes of estrogen biosynthesis, transport and metabolism have shown significant association with both gallstone disease and biliary tract cancer possibly by modulating hormone metabolism [18]. The IVS1-397C>T and IVS1-351A>G polymorphisms have been an important area of research in diseases such as osteoporosis [22], [38], [39] cardiovascular disease [40] and cancer [41]. A number of hypotheses for the functional significance of these polymorphisms have been reported in the literature. Given their location, 397 and 351 base pairs upstream from the start of exon 2, possible functional mechanisms include altered ESR1 expression by differential binding of transcription factors and influencing alternative splicing. Our in-silico studies further support the influence of genetic variants of estrogen receptor on gene transcription and splicing mechanisms. Thus, estrogen could enhance cholesterol cholelithogenesis by augmenting functions of estrogen receptors in the liver and gallbladder [42]. Considering the importance of hormonal receptors in gallbladder function, the hypothesized variants may result in cholesterol gallstones followed by chronic inflammation and further intense metaplasia, ultimately progressing into GBC which in turn may be categorized as the gallstone dependent pathway of estrogen receptors.

For progesterone receptor polymorphism, very little is known about the functional consequences by which *Alu* insertion in progesterone receptor protect individuals from gallbladder carcinoma and gallstones. PROGINS allele codes for PGR that consists of 306 bp Alu insertion in the G intron. *PGR* ins/del is in perfect linkage disequilibrium (D’ = 1.0) with V660L polymorphism (rs1042838) [43] (i.e. The insert-carrying allele (PGR I) exhibits higher mRNA stability and is transcribed to a more stable and transcriptionally active protein [44]. The PROGINS insertion allele has been reported as inversely correlated with risk of breast cancer [45], [46], [47] and endometriosis [48] in some populations [49], [50], [51]. It is believed that increased PGR may inhibit the mitogenic activity of insulin-like growth factors (IGFs), possibly through the regulation of Insulin-like growth factor-binding protein 1 (IBP-1) and thus influence cancer risk [52], [53], [54].

### Study Limitations

Although, sample size in the present study is sufficient to yield 80% power but it is limited in subgroup analysis. Therefore, study may require confirmation in larger cohorts. Because this is an association study, we cannot rule out the presence of possible linkage disequilibrium with other neighboring genes that might explain the significant association with gallbladder cancer phenotypes or adverse prognosis.

In conclusion, our study showed that *ESR1* IVS1-397C>T (rs2234693), *ESR1* IVS1-351A>G (rs9340799), *ESR2* -789 A>C (rs1271572) polymorphisms and their higher-order interactions may confer increased risk of gallbladder carcinoma, probably through gallstone mediated pathway.

## Methods

### Ethics Statement

The study protocol was approved by the institutional ethical committee of Sanjay Gandhi Post Graduate Institute of Medical Sciences (SGPGIMS), and the authors followed the norms of World’s Association Declaration of Helsinki. All the participants were provided with written informed consent for the study.

### Study Population

The present case control study recruited a total of 860 subjects, including 410 GBC patients which included 230 previous GBC cases [26], 230 gallstone patients (GS) and 220 healthy subjects. All unrelated subjects were of North Indian ethnicity. Patients were consecutively diagnosed between June 2006 and September 2011 at the Dept. of Gastro-surgery, Sanjay Gandhi Post Graduate Institute of Medical Sciences and Dept. of Surgical Oncology, CSMMU Lucknow, India. GBC was defined as tumor arising at the innermost (mucosal) layer and spreading through the visceral peritoneum (tissue that covers the gallbladder) and/or to the liver and/or to one nearby organ (such as the stomach, small intestine, colon, pancreas, or bile ducts outside the liver), and to nearby lymph nodes. Cancer diagnosis for all cases was confirmed by Fine Needle Aspirated Cell cytology (FNAC) and histopathology, yielding a response rate of 94%. Staging of cancer was documented according to the AJCC/UICC staging [55]. In gallstone disease, symptomatic gallstones were detected by transabdominal ultrasonography. The healthy controls were recruited from unrelated individuals free of any malignancy from general population. Individuals with silent gallstones detected by ultrasonography were excluded from the controls. The controls were frequency matched to patients for age (±5 years) and sex. At recruitment, informed consent was obtained from each subject and the information on demographic characteristics, such as sex, age and smoking habit, was collected by questionnaire.

### DNA Samples and Genotyping

On the basis of previous functional and epidemiological studies [17], we selected a total of 6 literature-defined functional polymorphisms in three important genes involved belonging to estrogen and progesterone receptors. Candidate single nucleotide polymorphisms (SNPs) were chosen based on the following: (a) the allele frequency of over five percent in published literature or databases [56] (b) validated allelic substitutions, and/or (c) functional changes linked with allelic substitution reported in the literature. These included three single nucleotide polymorphisms (SNPs) in estrogen receptor 1 (*ESR1*: IVS1-397C>T rs2234693, IVS1-351A>G rs9340799), and Ex4-122C>G (rs1801132) gene, two SNPs in the estrogen receptor 2 (*ESR2*:-789 A>Crs1271572, 1082 G>A rs1256049) gene and one SNP in progesterone receptor (*PGR*: ins/del rs1042838). Genomic DNA was extracted from 5 ml peripheral blood leukocytes according to standard salting out method [57]. The blood sample and the clinical details were collected from each participant at recruitment. The polymorphisms were genotyped using the PCR or PCR-restriction fragment length polymorphism method as described earlier by Lai *et al* [58] and Rowe *et al*. [59]. The digested PCR fragments were separated on polyacrylamide gel, stained with ethidium bromide and observed with ultraviolet imaging system (Bio-Rad Model). Genotyping was performed without knowledge of the case or control status. A 10% masked, random sample of cases and controls were tested twice by different laboratory personnel and the reproducibility was 100%.

### Statistical Analysis

Descriptive statistics were presented as mean and standard deviation [SD] for continuous measures while absolute value and percentages were used for categorical measures. The chi-square goodness of fit test was used for any deviation from Hardy Weinberg Equilibrium in controls. Differences in genotype and allele frequencies between study groups were estimated by chi-square test. Unconditional multivariate LR was used to estimate odds ratios [ORs] and their 95% confidence intervals [CIs] adjusting for age and sex. The ORs were adjusted for confounding factors such as age and gender. A two-tailed p-value of less than 0.05 was considered a statistical significant result. All statistical analyses were performed using SPSS software version 16.0 (SPSS, Chicago, IL, USA). Haplotype analysis was performed using SNPstatwww.snpstats.in [60].

Multifactor dimensionality reduction (MDR) method is non-parametric, genetic model-free method for overcoming some of the limitations of logistic regression (i.e. sample size limitations) for the detection and characterization of gene–gene interactions [61]. In MDR, multilocus genotypes are pooled into high risk and low risk groups, effectively reducing the genotype predictors from *n* dimensions to one dimension (i.e. constructive induction). The new one-dimensional multilocus genotype variable is evaluated for its ability to classify and predict disease status through cross-validation and permutation testing. The MDR software (version 2.0 beta8) was applied to identify high-order gene-gene interactions associated with GBC risk. In our study, the best candidate interaction model was selected across all multilocus models that maximized testing accuracy and the cross-validation consistency (CVC). Furthermore, validation of models as effective predictors of disease status was derived empirically from 1000 permutations, which accounted for multiple comparison testing as long as the entire model fitting procedure was repeated for each randomized dataset to provide an opportunity to identify false positives. The MDR permutation results were considered to be statistically significant at the 0.05 level. All the variables identified in the best model were combined and dichotomized according to the MDR software and their ORs and 95% CIs in relation to GBC risk were calculated. Finally, combined effect of the variables in the best model by the number of risk genotypes was evaluated using logistic regression analysis.

Classification and regression tree (CART) analysis was performed using the SPSS ver. 16 software to build a decision tree via recursive partitioning [26]. For the analysis, decision tree was created by splitting a node into two child nodes repeatedly, beginning with the root node that contains the total sample. Before growing a tree, we choose measure for goodness of split using Gini criteria, by which splits were found that maximize the homogeneity of child nodes with respect to the value of the target variable. After the tree was grown to its full depth, a pruning procedure was performed to avoid over fitting the model. Finally the risk of various genotypes was evaluated by using the logistic regression analysis. The ORs and 95% CIs were adjusted for age and sex, with treating the least percentage of cases as the reference.

### In Silico Analysis

The putative functional effects were determined in both coding and non-coding regions of ESR and ESR-2 gene by online web servers FASTSNP (http://fastsnp.ibms.sinica.edu.tw) and F-SNP http://compbio.cs.queensu.ca/F-SNP/ [62], [63] and in case of coding regions the effect on the protein structure was considered. The following features were used to identify the effect of SNPs in non-coding regions: Transcription factor binding sites (TFBS), Intron/exon border consensus sequences (splice sites), Exonic splicing enhancers (ESEs), and Triplex-forming oligonucleotide (TFO) target sequences.
